# Economic evaluation of a community health worker model for tuberculosis care in Ho Chi Minh City, Viet Nam: a mixed-methods Social Return on Investment Analysis

**DOI:** 10.1186/s12889-023-15841-2

**Published:** 2023-05-25

**Authors:** Luan Nguyen Quang Vo, Rachel Jeanette Forse, Jacqueline Tran, Thu Dam, Jenny Driscoll, Andrew James Codlin, Jacob Creswell, Kristi Sidney-Annerstedt, Vinh Van Truong, Ha Dang Thi Minh, Lan Nguyen Huu, Hoa Binh Nguyen, Nhung Viet Nguyen

**Affiliations:** 1 Friends for International TB Relief, 6th Floor, 1/21 Le Van Luong St., Nhan Chinh Ward, Thanh Xuan District, Ha Noi, Viet Nam; 2 Stop TB Partnership, Geneva, Switzerland; 3 Department of Global Public Health, WHO Collaboration Centre on Tuberculosis and Social Medicine, Karolinska Institutet, Solna, Sweden; 4 Pham Ngoc Thach Hospital, Ho Chi Minh City, Viet Nam; 5 National Lung Hospital, Ha Noi, Viet Nam

**Keywords:** Social Return on Investment, Economic evaluation, Mixed-methods, Community health workers, Tuberculosis, Active case finding, Viet Nam

## Abstract

**Background:**

There is extensive evidence for the cost-effectiveness of programmatic and additional tuberculosis (TB) interventions, but no studies have employed the social return on investment (SROI) methodology. We conducted a SROI analysis to measure the benefits of a community health worker (CHW) model for active TB case finding and patient-centered care.

**Methods:**

This mixed-method study took place alongside a TB intervention implemented in Ho Chi Minh City, Viet Nam, between October-2017 – September-2019. The valuation encompassed beneficiary, health system and societal perspectives over a 5-year time-horizon. We conducted a rapid literature review, two focus group discussions and 14 in-depth interviews to identify and validate pertinent stakeholders and material value drivers. We compiled quantitative data from the TB program’s and the intervention’s surveillance systems, ecological databases, scientific publications, project accounts and 11 beneficiary surveys. We mapped, quantified and monetized value drivers to derive a crude financial benefit, which was adjusted for four counterfactuals. We calculated a SROI based on the net present value (NPV) of benefits and investments using a discounted cash flow model with a discount rate of 3.5%. A scenario analysis assessed SROI at varying discount rates of 0-10%.

**Results:**

The mathematical model yielded NPVs of US$235,511 in investments and US$8,497,183 in benefits. This suggested a return of US$36.08 for each dollar invested, ranging from US$31.66-US39.00 for varying discount rate scenarios.

**Conclusions:**

The evaluated CHW-based TB intervention generated substantial individual and societal benefits. The SROI methodology may be an alternative for the economic evaluation of healthcare interventions.

**Supplementary Information:**

The online version contains supplementary material available at 10.1186/s12889-023-15841-2.

## Background

Tuberculosis (TB) remains a global public health challenge [1]. About 10 million people worldwide contracted TB in 2019 and almost half a million individuals developed multidrug-resistant TB (MDR-TB) [2]. Individuals with TB often face barriers to accessing and completing treatment throughout the cascade of care [3]. Pre-treatment barriers can be logistical, socioeconomic or sociocultural in nature, often working in unison to cause patients to delay or forego health-seeking [4, 5]. After enrollment, the direct and economic costs of productivity loss from TB treatment constitute major burdens [5]. Drug resistance severely exacerbates burdens related to disease and treatment, and associated risk of unfavorable outcomes [6]. TB survivors often carry long-term medical and social sequelae and elevated risk of chronic recurrence [7, 8]. The impoverishing effects of TB on affected households have been widely documented [9]. The average cost of TB care was equivalent to 58% of reported annual individual income and 39% of reported household income. These costs as a proportion of income were higher for people with low income and for those with MDR-TB [10]. These economic disincentives throughout the care cascade contribute to loss to follow-up (LTFU), chronic morbidity, development of drug resistance and continued community transmission [11, 12]. Realizing that progress in fighting TB needs to be measured by more than clinical outcomes, the WHO included the elimination of catastrophic cost, defined as incurring TB-related costs over 20% of annual household income, as one of three core performance indicators in its End TB Strategy [13–15].

Global societal costs attributable to TB were estimated to be US$4 billion each year in direct healthcare and US$12 billion in lost productivity [16]. Across 135 low- and middle-income countries (LMIC), total spending on TB increased 3.9% per year between 2000 and 2017, with overall government spending for detected and missed cases reaching US$6.9 billion in 2017 [17]. From the health system perspective, the average treatment cost was US$6,667 per case of drug-susceptible TB (DS-TB) and US$46,219 per case of MDR-TB [18].

Given these costs, many studies have documented the cost-effectiveness of TB interventions [19–23]. Targeted TB interventions were cited to produce a return of US$56 per dollar spent with benefit-cost ratios (BCR) ranging from 11–192:1 [24]. A modelling study found active case finding (ACF) to be a cost-effective tool in India, China, and South Africa as the cost per disability-adjusted life year (DALY) averted was lower than the per capita gross domestic product [25]. Besides ACF, engagement of all stakeholders in the community [22, 26, 27] or private sector [28–30], is another strategy commonly identified as cost-effective. Regarding MDR-TB, the BCR for ACF as well as diagnosis and treatment of MDR-TB were cited to be US$32 (6–47:1) and US$2 (0–23:1), respectively [24]. Concordantly, a systematic review found MDR-TB treatment to be a highly cost-effective intervention in LMICs [31].

Despite much evidence, there is large heterogeneity in TB intervention valuations, including methodology, design and perspective [32]. Health economic analyses use basic methods including reporting of costs, [33] and cost minimization [34, 35] expressed as lowest cost to achieve one empirical effect, e.g., TB case detection, and BCRs. More sophisticated procedures employed cost-utility analyses, [23] defined as costs to achieve unified effects expressed as Quality-adjusted life-year (QALY) or DALY, and cost-effectiveness analyses [36, 37] estimating the incremental costs to achieve an incremental empirical effect, e.g., additional costs per additional successful TB treatment. Designs can be empirical (retrospective case analyses, randomized trials) or mathematical (decision-tree and Markov simulation models) [38]. These analyses can assume patient [39], health system [40], and sometimes, societal perspectives [41].

An alternative economic evaluation method to achieve this comprehensive perspective involves estimating social return on investment (SROI), [42] defined as the monetary value generated for society for every unit of money invested. An SROI is a simultaneous valuation of personal, social and community outcomes [43]. By capturing the benefit and costs from multiple stakeholder perspectives, SROI offers a more comprehensive assessment of an intervention’s net benefit for society as a whole and thus a more compelling analysis for policy-makers. There is a growing body of evidence employing the SROI methodology in various settings and for diverse development and healthcare interventions with demonstrated value in program evaluation [44–47]. However, evaluations employing SROI for TB interventions remain scarce as our literature review found no such published studies. A systematic review in 2019, found only eight peer-reviewed SROI studies for health, only one of which was conducted in a LMIC [48].

In 2020, there were 172,000 persons with incident TB, including 8,400 persons with MDR-TB, and 10,400 TB-related deaths in Viet Nam [49]. Nevertheless, the government remains committed to ending TB by 2030 [50]. The National TB Control Programme (NTP) identified a greater engagement of community health workers (CHW) as a key strategy to achieve this goal [51]. Between 2017 and 2019, Friends for International TB Relief piloted a TB intervention in Ho Chi Minh City (HCMC), Viet Nam, called Proper Care. The project employed CHW to systematically screen vulnerable populations and provide support to patients on treatment. Prior evaluations reported this intervention model increased case detection [52] as well as reduced LTFU [53] and catastrophic cost incurrence [54]. The current study evaluated the economic impact of Proper Care via the SROI method to offer further evidence to national stakeholders for intensified intervention towards ending TB.

## Methods

### Study design

This was a mixed-methods study to construct an evaluative SROI analysis with a 5-year time-horizon on the economic benefit of a CHW-based TB intervention.

The planning and design of this mixed-method study was developed based on the strategies and procedures outlined by Creswell [55]. Accordingly, the transformative mixed-method strategy was selected whereby a theoretical lens was applied based on a rapid literature review followed by the qualitative research to apply an inductive approach and quantitative methods to expand findings from the prior two strategies.

The literature review framed the health economic evaluation and qualitative research identified and synthesized pertinent stakeholders and material value drivers. Our quantitative methods encompassed the development of the model components and calculation of the discounted cash flow (DCF) to arrive at the SROI estimate.

This study was conducted according to the guidelines and principles of the Consolidated Health Economic Evaluation Reporting Standards (CHEERS) statement [56].

### Intervention

The intervention was additional to routine TB program activities and took place from Oct-2017 to Sep-2019 in three districts of HCMC, Viet Nam, with a population of 1,465,819. CHWs systematically screened household and first-degree contacts of active TB patients and key vulnerable populations, and then referred persons with symptoms suggestive of TB or collected and transported sputum to the District TB Unit (DTU) for further evaluation. Health-seeking persons received a study-subsidized chest X-ray and free sputum testing as per national treatment guidelines [57]. Individuals diagnosed with TB were initiated on appropriate treatment according to NTP guidelines. CHWs provided adherence counseling and psychosocial support to all patients treated at the DTU. For destitute families, CHWs sometimes provided self-financed nutrition support and transport to the DTU for follow-up consultations. CHWs were supported by a site coordinator responsible for supervision and coordination. The intervention was previously described [53, 58]. In total, the intervention involved approximately 20 public health staff at the DTUs and provincial lung hospital and 151 CHWs and coordinators in the community. The total number of persons with TB engaged before and during the intervention was 7,776 and the number of family members and other vulnerable persons screened for TB by the intervention was 38,130 (Supplementary information – TB-SROI Model).

### Model development procedures

**Fig. 1:**
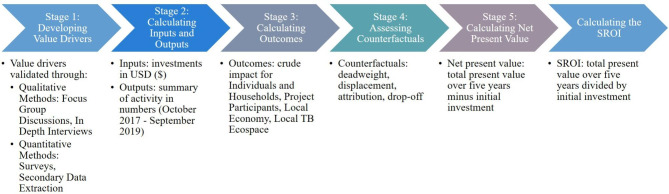
Conceptual framework of the stages of the evaluative SROI analysis

The SROI analysis contained five stages based on the guidelines and principles developed by Social Value UK [59]: (1) identifying stakeholders and value drivers, (2) calculating inputs and outputs, (3) calculating crude social returns on investment, (4) incorporating counterfactuals, and (5) calculating net present value (NPV) and SROI (Fig. 1). Value drivers are defined as sources of value creation or destruction. Net present value is defined as the current discounted value of future cash flows after subtracting any investments.

Stage 1: We identified hypothetical stakeholders and value drivers based on a rapid literature review [60, 61]. In summary, this rapid review aimed to assess existing knowledge on the application of SROIs for valuation of interventions in health and TB using systematic review methods, but conceding breadth or depth by limiting aspects of the process to compensate for time limitations [62]. The detailed search methodology is provided in the Supplementary information – Rapid literature review. To validate the stakeholders and value drivers, we conducted two focus group discussions (FGD, n = 15) and 14 in-depth interviews (IDI). Participants were purposively sampled to achieve information power [63]. Interviews were conducted using a topic guide about the benefits of the intervention, challenges faced, and potential negative impacts of the intervention on individuals and society. Audio recordings were collected and transcribed in the language of the interview (Vietnamese or English). The audio and transcriptions were compared to ensure accuracy. Interviews in Vietnamese were professionally translated and independently verified. Inconsistencies in the translation were discussed among a group of translators until consensus was reached. Qualitative data were coded and analyzed in Dedoose v*8.3.17* (SocioCultural Research Consultants; Los Angeles, CA). We used content framework analysis for qualitative research [64, 65] to validate the stakeholders and value drivers.

Stage 2: After identification of pertinent stakeholders and material value drivers, we calculated the associated inputs and outputs. Inputs were financial and non-monetary stakeholder resources to enable interventions such as activity implementation budgets and self-financed payments by CHWs and site coordinators for patient support. All costs were converted to US$ at an average rate of US$1 = VNĐ22,921 (2017–2018, oanda.com). Outputs were a quantitative summary of each of the value drivers by stakeholder. Sample outputs included the additional TB patients detected and linked to treatment through ACF, number of project staff hired and compensated for their efforts, and amount of additional funding obtained throughout the intervention period. The number of additional persons with TB detected (additionality) was determined based on a comparison with operational and clinical data from the baseline period (2016) [66]. Quantitative inputs and outputs for each value driver were derived from primary and secondary data sources. One primary data source entailed a quantitative survey informed by the qualitative research activities (provided in the supplemental information). This survey included 13 questions and was fielded by study staff among 11 project beneficiaries through in-person/phone conversations. The survey collected data on previous and current monthly income, average length of routine and after-hour work, and material out-of-pocket costs for the intervention. Additional primary sources included the intervention’s operational data repository, routine surveillance data from the NTP, human resource and payroll registers, and project accounting records. Secondary data sources included published scientific literature and ecological databases.

Stage 3: The operational outputs of each value driver were monetized to obtain a crude financial return. The monetization process used financial proxies to arrive at price assumptions obtained from primary and secondary sources as described above. Financial proxies assign a monetary value to an operational indicator or activity. An example of a financial proxy is the minimum wage as a proxy for average income per patient per year to estimate the value of individual benefit for persons detected and cured of TB. Monetary benefits were positive and negative, and encompassed beneficiary, health system and societal perspectives. Monetization procedures were tailored to previously identified relevant stakeholders: (1) TB-affected patients and households; (2) site coordinators and CHWs; (3) NTP; (4) municipal government; and (5) Viet Nam TB ecosystem. In this case, ecosystem is defined as the overall amount of available funds for TB care and prevention in Viet Nam.

For TB-affected patients and households, we calculated the additional income earned after being cured of TB for persons who were detected and linked to treatment through ACF. We further quantified the reduction of mortality rates in the ACF and Passive Case Finding (PCF) cohorts in monetary terms. The price assumption for the additional income generated was based on the minimum wage legislation for the intervention area [67]. Lastly, we also included pre-treatment cost avoidance through early detection via ACF using values reported by the Viet Nam national patient cost survey [39].

Crude financial returns on investment for site coordinators and CHWs were calculated by summation of the salaries (fixed compensation) and incentives (variable compensation) paid over the course of the project. The model incorporated a negative impact from one of the field staff contracting TB. To quantify this return in monetary terms, we used household costs and health care system costs for DS-TB treatment from published scientific literature [39].

First, returns generated for the NTP included the fixed and variable stipends paid to district and provincial staff to compensate for time and effort dedicated to the study that were incremental to their routine responsibilities. Other positive returns such as increased capacity building and awareness about TB and their role as well as improved performance and ranking within the government’s performance appraisal system were excluded according to the SROI principles of materiality and not over-claiming. Based on the same principle, we did not incorporate negative returns such as detraction from the daily responsibilities resulting from participation in the study. Second, the impact of the NTP included MDR-TB treatment cost avoidance calculated based on the difference in treatment outcomes of LTFU, treatment failure and transfer out measured during the intervention in comparison to the pre-intervention baseline period. Clinical assumptions and financial proxies for the monetization of the MDR-TB cost avoidance were obtained from published literature [33, 68].

The municipal government benefitted from the macroeconomic value of TB-related mortalities averted. We employed the Value of Statistical Life (VSL) method to calculate the VSL-Year (VSLY) based on average life expectancy in Viet Nam. To localize the US-based VSLY results, we employed the benefits-transfer method at unit elasticity to obtain VSLYs for Viet Nam [69]. The benefits-transfer method relies on the GDP ratio of two countries and has been used to translate VSL between high-income countries with reliable data from hedonic wage studies to low- and middle-income countries that tend to lack these data [70]. Macroeconomic productivity loss was estimated for persons with TB detected through ACF or PCF, who completed treatment during the study period.

To assess the benefits to the Viet Nam TB ecosystem, we counted all additional resources mobilized during the study period that were based on the Proper Care model. These resources represent the leverage gained from the initial investment in the Proper Care project that resulted in the co-financing from two additional funding mechanisms.

Stage 4: The SROI method integrates four counterfactuals to adjust crude financial returns from the previous stage. These adjustment parameters include: (1) deadweight; (2) displacement; (3) attribution; and (4) drop-off [71]. Deadweight represents the benefits that would have arisen irrespective of the intervention. Displacement represents any activity and associated economic gains the intervention displaced through its activities. Attribution represents the amount of impact that can be attributed to project activities and contributions. Drop-off represents a decline in recurring value generated from the project over time [59].

Stage 5: A DCF model was used to calculate the NPV of the monetary benefits and costs over a 5-year time-horizon to appropriately measure the impact of mortality avoidance. Thus, even though several value drivers would generate impact beyond the evaluation horizon, this impact was truncated after a 5-year projection to avoid overestimation of the impact. The discount rate to estimate the NPV was 3.5% based on the 2017 reported inflation rate in Viet Nam [72]. A sensitivity analysis tested the effect of applying a discount rate ranging from 0 to 10% on NPV and SROI.

### Ethical considerations

The study was conducted in accordance with the Helsinki Declaration (7th Revision) as well as the guidelines and principles of the Consolidated Health Economic Evaluation Reporting Standards (CHEERS) statement. We obtained ethical approval for the study from the Institutional Review Board of the Pham Ngoc Thach Hospital in Ho Chi Minh City (129/HDDD-PNT). All participants provided informed, written consent and all data were anonymized prior to analysis.

## Results

### Stakeholders and value drivers

**Fig. 2:**
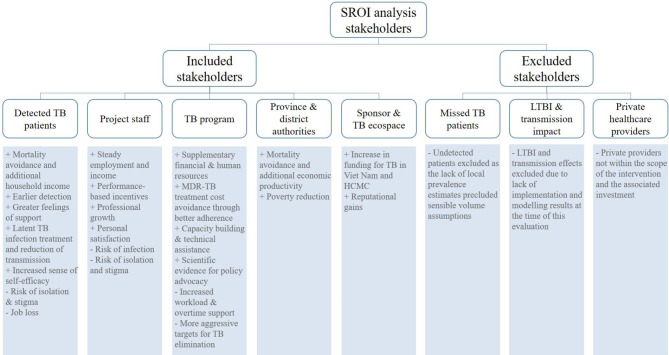
Stakeholders and hypothesized value drivers

Our rapid literature review resulted in 65 search results of which all titles were reviewed. We excluded 12 studies not relevant to our study or that were published prior to the year 2000, and read the abstracts of the remaining 53 studies. Based on these abstracts, we reviewed 12 manuscripts in detail. This yielded eight stakeholders and 25 positive and negative value drivers of the intervention (Fig. 2). Based on data gathered through the FGDs and IDIs, we narrowed the scope to five relevant stakeholders and 14 material value drivers. Table 1 presents a summary and representative quotations of the qualitative data for the validation of the hypothesized value drivers by individual stakeholder groups, while Table 2 presents a summary of the perceived benefits and costs by stakeholder groups.

**Table 1:** Perceived positive and negative value drivers of the intervention

Stakeholders	Perceived positive and negative value drivers identified by Stakeholders	Representative quote on the perceived positive value of the intervention	Representative quote on the perceived negative value of the intervention	
TB patients (n = 11)	• CHWs increase feelings of support and care through counselling • Encouragement from CHWs increased self-efficacy and motivation to complete treatment • Prevention of family or community from TB infection • Anticipated and/or experienced isolation and stigma	*“I had to try to follow the treatment. Luckily, I had the help from my healthcare worker. She encouraged me and helped me receive my medicine. In general, I was very happy and even went to hug her and said thank you. Without her, I don’t know what I would have done.” – Male, TB Patient, Painter*	*“I was afraid that people would avoid me since this is a communicative disease. Many people have knowledge about TB and are okay with it. But for people who do not know about TB, they are probably scared.”- Male, TB Patient, Silversmith*	
Site coordinators & CHWs (n = 9)	• Earlier disease detection • More access to healthcare services • Increased sense of personal satisfaction due to positive patient outcomes • Creation of community and strengthened interpersonal relationships • Lack of patient trust in the health care system	*“My healthcare worker lit a flame in me, she encouraged us to place more heart in our jobs, we felt excitement in our work. We feel happy when working with patients and creating trust. I have worked for this for nearly twenty years, and I love my job.” – Female, Community Health Worker*	*“The most difficult thing is trust. For example, there was a case of a drug-resistant TB patient who had a positive result, but he did not believe that he suffered from TB. He did not agree to treatment even though the TB counsellor visited his house and a healthcare worker talked with him.” – Male, Community Health Worker*	
TB program (n = 3)	• Direct support for ACF activities • Strengthened collaborations between National and Provincial TB programs and local governments • CHWs supported programs and increased collaboration • Heavier workload and need for overtime support	*“The force of community health workers has worked so far and I still highly appreciate them and consider them as the important bridge between the project, people with TB, clinics and units.” – Male, Head District TB Unit Officer 1*	*“It is not just that we work extra hours, but the intensity of screening will also be heavier. With the program, patients get chest x-rays and some days there may be up to 30. Therefore, there is more work to do.” – Male, Head District TB Unit Officer 2*	
Provincial and District Government (n = 3)	• Direct support for ACF activities • Broader community and household reach • Ability to target high risk groups • Fear and lack of trust of the government from patients	*“Since the Proper Care program started, we are more active, we find cases actively at our unit. For example, each year, we detect about 100 TB patients, but the number of TB cases increased to 150 patients last year. – Male, District Health Center Director 1*	*“Our activities make people afraid of being taken advantage of due to the approach. For some difficult places, we have to invite leaders of the residential clusters. They will come along with us for safety reasons, moreover to avoid the negative exploitation of counsellors’ and health workers’ work.” – Male, District Health Center Director 2*	
Viet Nam TB Ecosystem (n = 3)	• Increase in funding for TB • Influence on domestic policy • Improvement of corporate image and reputation • Communicating project mission fit to corporations	*“It really helped us to inform national policy. Back in the day, we didn’t really know what it’s like on the ground. Now, we have someone who really understands the details to help us make an impact. – Male, Organization Country Director*	*“One of our challenges was really to convince our management that the Proper Care project was worth investing in. There’s a pot of money that was available to us, and we were all competing for it” – Female, Organization Department Senior Director*	
Abbreviations: ACF: Active Case Finding, CHW: Community Health Worker, TB: Tuberculosis				

**Table 2:** Key stakeholders and their perceived benefits and challenges

Stakeholders	Perceived benefits	Perceived challenges
TB patients	Patients noted that CHWs were the main benefit due to their ability to provide both tangible support (TB knowledge and counseling) as well as feelings of being supported and having a caring community. Encouragement from CHWs increased feelings of self-efficacy and motivated patients to prevent spread and complete treatment.	Isolation and stigma were the main challenges, in terms of the patients themselves, and in their perception of others. However, this value driver was indicated as a difficulty of the disease, not necessarily of the program.
Site coordinators & CHWs	The largest benefit project staff experienced for themselves was a sense of personal satisfaction when patients experienced positive health outcomes. Patients were viewed as friends or peers, creating a stronger sense of community.	A challenge faced by patients, which also translated to a challenge for project staff, was a lack of trust in the health care system. Staff encountered difficulties in contacting patients or convincing patients to obtain treatment at the district-level TB treatment facility.
TB Program	Direct support for ACF activities was mentioned as the greatest benefit. District-level TB healthcare providers noticed strengthened collaboration between the National TB Control Program, implementing partners, and the local government. CHWs were an essential bridge between stakeholders and provided consultation and advocacy for patients. The program provided direct support though GeneXpert tests, and this program support produced an increased number of TB patients detected and linked to treatment.	District-level TB healthcare providers felt burdened with an increased workload when being required to perform setup activities and manage community activities. The number of meetings, training, and reports were increased and many providers requested additional staff to assist with the workload.
Provincial and District Government	For government members, direct support for ACF activities was most beneficial, with an emphasis on the increase in concrete numbers such as households approached and individuals screened. They acknowledged the comprehensiveness of the program: approaching vulnerable groups, thorough data analysis, and tangible improvements.	A challenge was a fear and lack of trust of the government from TB patients, which made it difficult to reach patients and effectively communicate the aims of the program.
Viet Nam TB Ecosystem	Sponsors saw an improvement in their image, reputation, and partnerships. The project facilitated collaboration between all relevant groups which was mutually rewarding, and strengthened the credibility of the project.	During the project’s inception, the challenge was convincing funders that the project fit with the overall goals of the corporations. Through observing activities at the grassroots level and collaborating with local organizations, sponsors were able to change their mindsets surrounding the project.

Abbreviations: ACF: Active Case Finding, CHW: Community Health Worker, TB: Tuberculosis

Across stakeholder groups, participants identified the clinical, economic and epidemiological benefits provided to individuals and society as the intervention’s main value driver. Patients appreciated the socioeconomic and psychosocial support from site coordinators and CHWs to help them recover from the illness.

*“I tried to follow the treatment. Luckily, I had the help from my healthcare worker. She encouraged me and helped me receive my medicine. Overall, I was very happy and even went to hug her and said thank you. Without her, I don’t know what I would have done.” – Male, TB Patient, Painter*.

Site coordinators and CHWs expressed a strong personal satisfaction about simultaneously earning an income, expanding their knowledge, and contributing to society. These stakeholders also frequently mentioned earlier disease detection and increased access to healthcare services for their patients as one of the project’s key value propositions.

*“When participating in the project, I have a job to do, and have income for myself as well as more knowledge.” – Male, Site coordinator 1*.

TB program staff and municipal government representatives emphasized the benefit for citizens in their municipality and for society in general. They also highlighted their improved performance on public health indicators. Furthermore, they identified the strengthened collaboration between TB programs and local governments as a key benefit.

*“In terms of benefits, it is huge. If [the intervention] reduces the number of patients of this communicable disease, the transmission rate will decline. When the transmission rate decreases, the number of TB patients will decrease. The number of TB patients decreasing means the economic burden on a locality or district or city will decline. It’s one of the most important benefits of this program, because the lower the number of TB patients, the more healthy people can work and produce for society.” – Male, DTU Officer 1*.

Key benefits included increased funding for the Viet Nam TB ecosystem, greater value perception of the intervention and its investment, and gains in sponsor reputation. Moreover, the intervention facilitated a strong partnership between all relevant groups and elucidated grassroots realities to national actors.

*“It really helped us to inform national policy. Back in the day, we didn’t really know what it’s like on the ground. Now, we have someone who really understands the details to help us make an impact. – Male, NGO Country Director*.

A notable challenge was trepidation and experience of isolation and stigma as part of the disease as well as a lack of trust in the health care system. This lack of trust was underlined by the need for CHWs and study staff to dedicate additional time and resources to travel to patient homes and provide counseling to promote enrollment onto the government TB treatment program.

*“The most difficult thing is trust. There was a drug-resistant TB patient with a positive result, but he did not believe that he suffered from TB. He did not agree to treatment even though the TB counselor visited his house and a public health officer talked with him.” – Female, CHW*.

Another key concern among CHWs and patients was contracting the disease when fulfilling their responsibilities.

*“We talk so much about our job, our children said that you go to work for the community and take the disease home.” – Female, Site coordinator 2*.

### Social return on investment

**Fig. 3:**
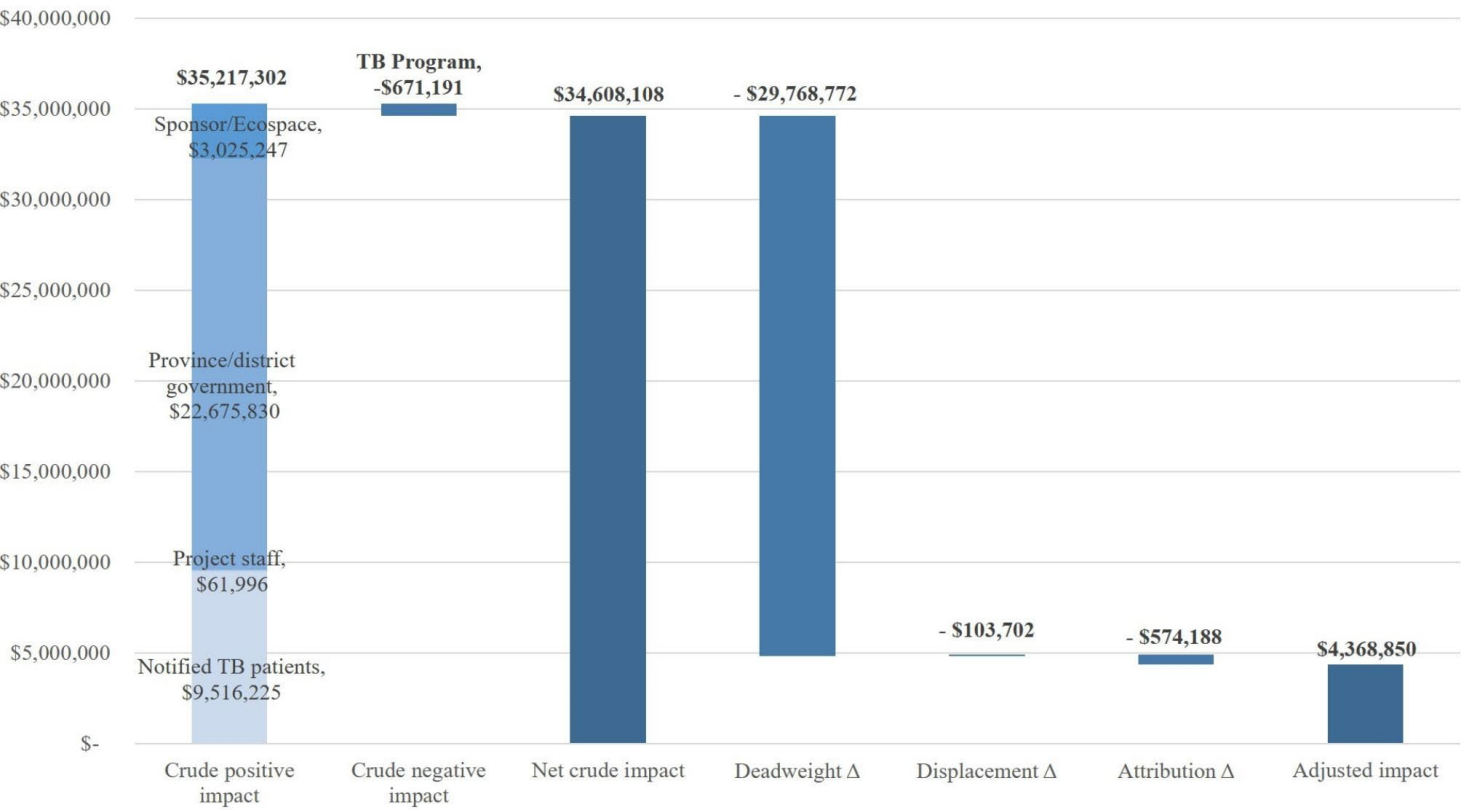
Adjustment of crude impact for counterfactuals

The total crude impact was $34,608,108 (Fig. 3). A large portion of the value ($9,516,225) was among treated TB patients, driven by the additional income generated from mortality avoidance ($8,971,527). The crude impact among site coordinators and CHWs overall was positive and estimated to be $61,996, primarily consisting of salaries and stipends. The crude impact for the overall TB program was negative, $-671,190, which was mainly driven by a net increase in LTFU (-$696,344) and higher estimated costs associated with drug-resistant TB management. The largest driver of crude impact with $22,675,830 was the macroeconomic benefit for municipal governments from the economic output generated by additional TB survivors. For the Viet Nam TB ecosystem, the crude impact was $3,025,247 from the additional resources mobilized with leverage from the initial investment. For a full breakdown of the crude returns on investment, please refer to the impact map provided in the supplementary information – TB-SROI Model.

After factoring in the counterfactuals, the adjusted impact was $4,368,850 (Fig. 3). This represents an 87% reduction from the initially-calculated crude impact. The largest adjustment ($-29,768,772) to our crude impact arose from deadweight, i.e., the benefit that would have been generated even in the absence of the investment represented by costs paid by the government for operating the NTP.

**Fig. 4:**
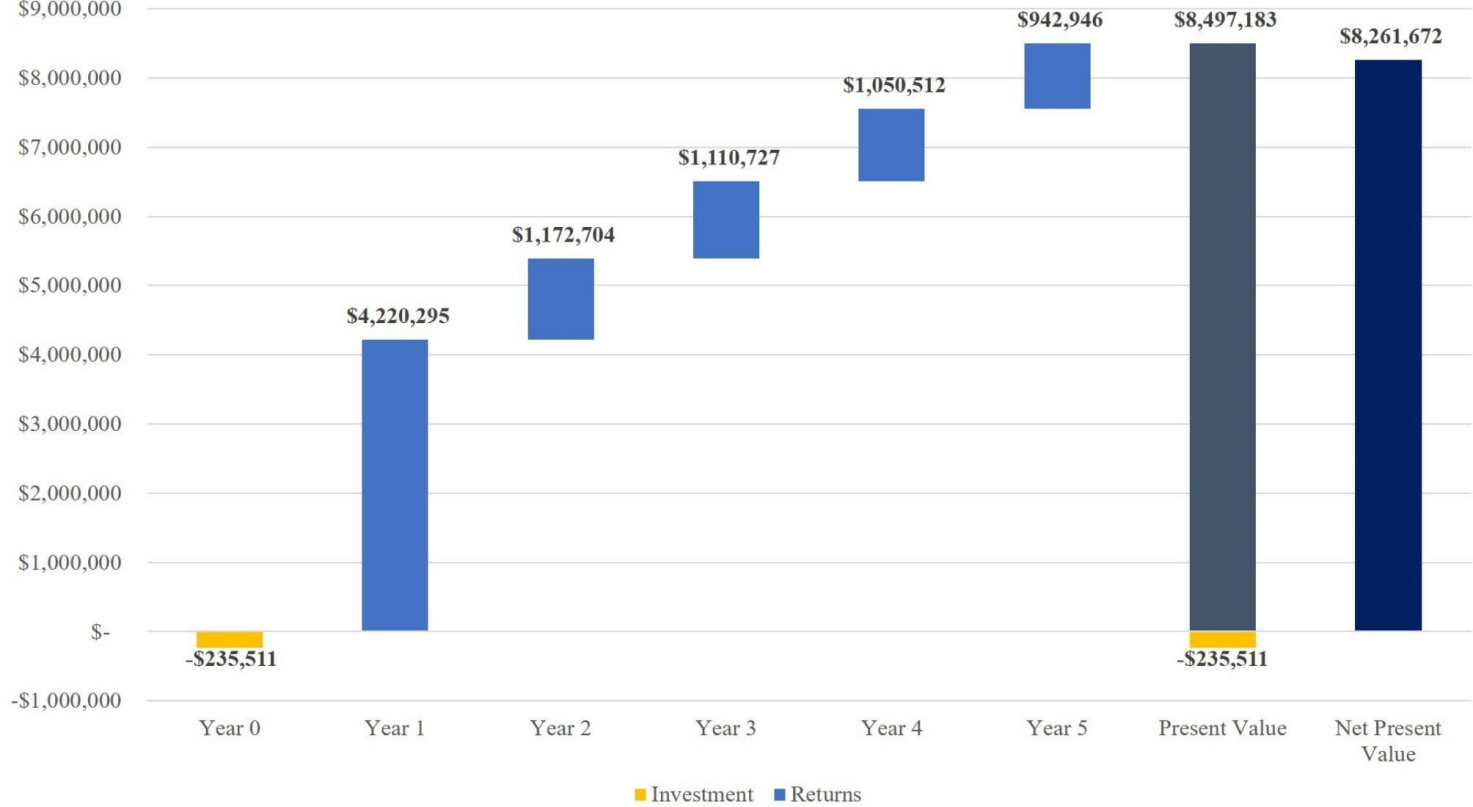
Discounted cash flow model results

Projecting this adjusted impact over the 5-year valuation window shows that the present value of returns from the intervention was $8,497,183 **(**Fig. 4). The largest benefits were gained in the first year with a return of $4,220,295. Returns diminished in subsequent years. Factoring in the initial investment of $-235,511 resulted in a NPV of $8,261,672, suggesting the SROI of the project was 3,608%. The sensitivity analysis showed the SROI ranged from 3,166%–3,900% for discount rates of 10% and 0%, respectively (supplemental information).

## Discussion

Our SROI analysis found that the investment in the Proper Care project created $36.08 in societal returns for every dollar spent. This result is concordant with existing evidence that ACF TB interventions using CHWs generate positive value for individual patients, local communities, and society [20, 73]. Furthermore, our SROI aligns with a study on TB in the Western Pacific Region that used a Solow model to estimate the return on investment for TB care in Vietnam and Laos, which ranged between $4-$49 per dollar spent [74].

The SROI measured on this project tended to be higher than results from other SROI analyses of general public health interventions. A systematic review of 26 evaluative analyses reported a median SROI of 509% (IQR: 253-729%).^75^ However, comparability with these studies is limited. Most had a time-horizon of one year and only six studies were from LMICs with none originating from Viet Nam or evaluating a TB-related intervention [75]. One key driver for the high SROI on this study may have been the inclusion of resources mobilized by leveraging this initial investment. This value driver was not included in any other studies and contributed to one-third of the value generated. Excluding this value driver would have lowered the estimated SROI of this intervention to 2,367%, which was in line with an analysis of a stigma reduction intervention among people living with HIV in Zambia (SROI = 2,120%) [76].

The highest value creation arose from additional income and economic output due to avoidance of TB-related mortality. This applies particularly to individuals with TB detected and successfully treated through ACF, as past studies have shown that many of these persons remain unreached by NTPs [77, 78]. These unreached persons also comprise the bulk of the estimated mortalities in the WHO’s annual global TB reports [2]. When combining the individual and economic gains from this intervention, the adjusted impact over the 5-year horizon constituted two-thirds of the returns. While this high proportion is clearly a function of the long time-horizon, it is concordant with prior modeling studies, which suggest that TB ACF can be highly cost-effective, especially when appropriately targeted, [36] but that short-term evaluations (1–2-year evaluation horizon) tend to underestimate its economic returns [25]. Benefits for individuals cured of TB include prolonged life, while and economic activity over the time-horizon evaluated represents a value to society.

A critical success factor of this project was the involvement of CHWs [79]. There is extensive evidence for the effectiveness of CHW-based models for TB ACF [80–82]. Similarly, studies in various settings reported CHWs were a critical component of patient- and person-centered care [83, 84]. Thus, CHW models were cited as a cost-effective way to provide healthcare services [20, 85]. By this intervention, these CHWs fulfilled the dual purpose of raising case detection and successfully supporting those patients to complete treatment. Therefore, the CHW model may have helped catalyze the high value creation from mortality avoidance.

This SROI evaluation had several limitations. First, given the operational research nature of the intervention and this study, we were unable to capture the effect of externalities such as a parallel private sector engagement project implemented in one of the intervention districts. Additionally, the limited geographic scope and distinct package of interventions limited the generalizability of the quantified benefits and SROI. Nevertheless, using SROI to evaluate the impact of our intervention was an effective way of measuring individual, health system and societal returns. This model also accounted for negative value drivers and placed a strong emphasis on adjusting for counterfactuals and discounting. Adjusting crude impact for counterfactuals was a particularly critical step of the analysis to avoid overestimation of the SROI. Specifically, these adjustments reduced the final impact to one-eighth of the crude value. This was mainly driven by deadweight among passively-detected cases that would have been successfully treated by the NTP irrespective of the intervention. The existing government structure contributed to most of the deadweight.

Another strength of this study was the combination of data sources and use of quantitative and qualitative methods. Particularly the latter enabled the discussion and refinement of value drivers with stakeholders, which were concordant with later studies analyzing the facilitators and barriers of ACF and patient support interventions [86]. Lastly, our methodology also generated information on the personal resources consumed by the intervention, which is uncommon in program evaluations [87]. The combination of these efforts yielded a deeper analysis to raise confidence in the positive, negative, and incremental value of an intervention.

## Conclusions

The SROI analysis shows that investment in CHW-supported ACF and TB patient support interventions generated substantial returns for a wide range of stakeholders. With its comprehensive perspective and conservative valuation, the SROI methodology may offer a viable alternative for economic evaluation of public health intervention that warrants further investigation. However, as with other benefit-cost analyses, the associated shortcomings should also be well considered prior to its application.

## Electronic supplementary material

Below is the link to the electronic supplementary material.

Supplementary Material 1

Supplementary Material 2

Supplementary Material 3

Supplementary Material 4

## Data Availability

The data that support the findings of this study are available from the Viet Nam National TB Control Program and Pham Ngoc Thach Hospital upon reasonable request to the corresponding author LNQV, but restrictions apply to the availability of these data.

## Abbreviations

ACF: Active Case Finding
AFB: Acid-fast Bacilli
CHW: Community Health Worker
CI: Confidence Interval
CXR: Chest X-ray
DTU: District TB Unit
EP: Extra-pulmonary
GEE: Generalized Estimating Equation
HCMC: Ho Chi Minh City
IRR: Incidence Rate Ratio
ITS: Interrupted Time Series
NNS: Number Needed to Screen
NTP: National TB Control Program
PBI: Performance-based Incentive
PNTH: Pham Ngoc Thach Provincial TB Hospital
TB: Tuberculosis
WHO: World Health Organization
