# Supplementary material for: Economic evaluation of a community health worker model for tuberculosis care in Ho Chi Minh City, Viet Nam: a mixed-methods Social Return on Investment Analysis
Source: BMC Public Health. 2023 May 25;23:945. doi: 10.1186/s12889-023-15841-2 (PMC10210363; doi:10.1186/s12889-023-15841-2)
Supplement: Supplementary file 1 — Supplementary Material 1 [file 12889_2023_15841_MOESM1_ESM.docx]

**CHEERS checklist—Items to include when reporting economic evaluations of health interventions**

| **Section/item** | **Item No** | **Recommendation** | **Reported on page No/ line No** |
| --- | --- | --- | --- |
| **Title and abstract** | | | |
| Title | 1 | Identify the study as an economic evaluation or use more specific terms such as “cost-effectiveness analysis”, and describe the interventions compared. | Lines 1-3 |
| Abstract | 2 | Provide a structured summary of objectives, perspective, setting, methods (including study design and inputs), results (including base case and uncertainty analyses), and conclusions. | Lines 24-46 |
| **Introduction** | | | |
| Background and objectives | 3 | Provide an explicit statement of the broader context for the study. | Lines 47-108 |
|  |  | Present the study question and its relevance for health policy or practice decisions. | Lines 108-110 |
| **Methods** | | | |
| Target population and subgroups | 4 | Describe characteristics of the base case population and subgroups analysed, including why they were chosen. | Lines 122-131;  Lines 137-147;  Lines 170-171 |
| Setting and location | 5 | State relevant aspects of the system(s) in which the decision(s) need(s) to be made. | Lines 121-122 |
| Study perspective | 6 | Describe the perspective of the study and relate this to the costs being evaluated. | Lines 168-169 |
| Comparators | 7 | Describe the interventions or strategies being compared and state why they were chosen. | Lines 103-108;  Lines 122-131 |
| Time horizon | 8 | State the time horizon(s) over which costs and consequences are being evaluated and say why appropriate. | Lines 121-122;  Lines 212-215 |
| Discount rate | 9 | Report the choice of discount rate(s) used for costs and outcomes and say why appropriate. | Lines 215-216 |
| Choice of health outcomes | 10 | Describe what outcomes were used as the measure(s) of benefit in the evaluation and their relevance for the type of analysis performed. | Lines 173-204 |
| Measurement of effectiveness | 11a | *Single study-based estimates:*Describe fully the design features of the single effectiveness study and why the single study was a sufficient source of clinical effectiveness data. | Lines 92-97; Lines 205-217 |
|  | 11b | *Synthesis-based estimates*: Describe fully the methods used for identification of included studies and synthesis of clinical effectiveness data. | not applicable |
| Measurement and valuation of preference based outcomes | 12 | If applicable, describe the population and methods used to elicit preferences for outcomes. | not applicable |
| Estimating resources and costs | 13a | *Single study-based economic evaluation:* Describe approaches used to estimate resource use associated with the alternative interventions. Describe primary or secondary research methods for valuing each resource item in terms of its unit cost. Describe any adjustments made to approximate to opportunity costs. | Lines 115-117;  Lines 137-165 |
|  | 13b | *Model-based economic evaluation:*Describe approaches and data sources used to estimate resource use associated with model health states. Describe primary or secondary research methods for valuing each resource item in terms of its unit cost. Describe any adjustments made to approximate to opportunity costs. | not applicable |
| Currency, price date, and conversion | 14 | Report the dates of the estimated resource quantities and unit costs. Describe methods for adjusting estimated unit costs to the year of reported costs if necessary. Describe methods for converting costs into a common currency base and the exchange rate. | Lines 121-122;  Lines 151-152 |
| Choice of model | 15 | Describe and give reasons for the specific type of decision-analytical model used. Providing a figure to show model structure is strongly recommended. | Lines 90-95;  Lines 98-100;  Figure 1 |
| Assumptions | 16 | Describe all structural or other assumptions underpinning the decision-analytical model. | Lines 133-136;  Lines 148-165;  Lines 173-204;  Supplemental information: Impact map |
| Analytical methods | 17 | Describe all analytical methods supporting the evaluation. This could include methods for dealing with skewed, missing, or censored data; extrapolation methods; methods for pooling data; approaches to validate or make adjustments (such as half cycle corrections) to a model; and methods for handling population heterogeneity and uncertainty. | Lines 205-217;  Supplemental information: Impact map |
| **Results** | | | |
| Study parameters | 18 | Report the values, ranges, references, and, if used, probability distributions for all parameters. Report reasons or sources for distributions used to represent uncertainty where appropriate. Providing a table to show the input values is strongly recommended. | Lines 224-228;  Figure 2; Tables 1 & 2;  Lines 274-293;  Supplemental information: Impact map |
| Incremental costs and outcomes | 19 | For each intervention, report mean values for the main categories of estimated costs and outcomes of interest, as well as mean differences between the comparator groups. If applicable, report incremental cost-effectiveness ratios. | Lines 274-283;  Lines 288-290;  Figures 3 & 4; |
| Characterising uncertainty | 20a | *Single study-based economic evaluation:* Describe the effects of sampling uncertainty for the estimated incremental cost and incremental effectiveness parameters, together with the impact of methodological assumptions (such as discount rate, study perspective). | Lines 284-287;  Lines 291-293 |
|  | 20b | *Model-based economic evaluation:*Describe the effects on the results of uncertainty for all input parameters, and uncertainty related to the structure of the model and assumptions. | Not applicable |
| Characterising heterogeneity | 21 | If applicable, report differences in costs, outcomes, or cost-effectiveness that can be explained by variations between subgroups of patients with different baseline characteristics or other observed variability in effects that are not reducible by more information. | not applicable |
| **Discussion** | | | |
| Study findings, limitations, generalisability, and current knowledge | 22 | Summarise key study findings and describe how they support the conclusions reached. Discuss limitations and the generalisability of the findings and how the findings fit with current knowledge. | Lines 295-300;  Lines 305-309;  Lines 327-331 |
| **Other** | | | |
| Source of funding | 23 | Describe how the study was funded and the role of the funder in the identification, design, conduct, and reporting of the analysis. Describe other non-monetary sources of support. | Information provided via the submission system, in the cover letter and the title page |
| Conflicts of interest | 24 | Describe any potential for conflict of interest of study contributors in accordance with journal policy. In the absence of a journal policy, we recommend authors comply with International Committee of Medical Journal Editors recommendations. | Lines 365-371;  Information also provided via the submission system and the title page |

For consistency, the CHEERS statement checklist format is based on the format of the CONSORT statement checklist
