# Supplementary material for: Economic evaluation of a community health worker model for tuberculosis care in Ho Chi Minh City, Viet Nam: a mixed-methods Social Return on Investment Analysis
Source: BMC Public Health. 2023 May 25;23:945. doi: 10.1186/s12889-023-15841-2 (PMC10210363; doi:10.1186/s12889-023-15841-2)
Supplement: Supplementary file 2 — Supplementary Material 2 [file 12889_2023_15841_MOESM2_ESM.docx]

**Supplementary Information**

*Supplemental methods*

Focus group discussion topic guide

**Participants**

- Focus group discussion 1
  - Notified TB patients detected through active case finding activities of the project
  - Notified TB patients detected through passive case finding at the District TB Units
- Focus group discussion 2
  - Project support staff
  - Site Coordinators (SCs)
  - TB Counselors
  - Community Health Works (CHWs)
  - District TB Unit (DTU) and commune-level TB officers
  - National TB Program (NTP) Representatives
  - Province/District
  - Donors/Sponsors
- Additional focus group discussions as needed

**Objectives**

- To explore the social and non-monetary benefits that Proper care brings to TB patients
- To gather poignant quotes from TB patients on Proper care
- To inform the SROI

**Introduction**

- Introduce FIT and study; confidentiality; timing

*Good afternoon. My name is ____ and this is my colleague ___. We are from FIT. Thank you for coming. A focus group is a relaxed discussion.*

*We are here today to talk about your experiences with the Proper care program. The purpose is to get your perceptions on how the program has benefitted you and society, especially in non-monetary ways.*

*There are no right or wrong answers. You can disagree with each other and you can change your mind. I would like you to feel comfortable saying what you think and how you really feel.*

**Procedure:**

*_____ will be taking notes and tape recording the discussion so that I do not miss anything you have to say. I explained these procedures to you when we set up this meeting. As you know everything is confidential. No one will know who said what and we ask that nothing said here leaves this room today. You can decline to answer any question you do not feel comfortable answering and there will be no consequence for you. You will receive all the care and treatment you need at the health facility as usual and you will not lose any benefit that you are entitled to such as receiving care and support that is provided at the clinic. All identification of participants will be anonymized,*

*I want this to be a group discussion, so feel free to respond to me and to other members in the group without waiting to be called on. However, I would appreciate it if only one person did talk at a time. The discussion will last approximately 90 minutes. There is a lot I want to discuss, so at times I may move us along a bit.*

**Life Background Intake Sheet for Focus Group Discussion Participants**

**TB Patients**

1. Family Background
   1. Where were you born?
   2. Who lives in your household?
2. School/education
   1. What is your highest education level?
   2. Where did you go to school?
3. Work history
   1. What is your current work?
   2. How long have you been working?
   3. What other types of jobs have you had?

**Public Health Officials and Non-Governmental Staff**

1. School/education
   1. What is your highest education level?
   2. Where did you go to school?
2. Work history
   1. How long have you been working?
   2. What is your current work?
   3. What position do you currently hold?
   4. How long have you held this position?
   5. What other positions/programs have you worked with prior to this position?

**Participant Introduction (5 minutes)**

*Now, let's start by everyone sharing their name, what they do, how long they’ve been diagnosed with TB (if in FGD with TB patients), and how long they’ve been in/worked for the Proper care program.*

**Life/Work Background (5 minutes)**

Use this section to get a sense of the work that the staff does not already collected on intake sheet. For TB patients, use this section to explore more detailed circumstances of the TB patients and get a sense of their life and their social network that is not already collected on intake sheet.

*For TB patients:*

- Family relationships
  - Probes: How often do you interact with family?
- Community relationships
  - Probes: What social networks do you have outside of your family?

*For public health officers and non-governmental staff:*

- Work
  - Probes: What is your current role? What are your main responsibilities?

**Experience with TB (10 minutes)**

Use this section to explore their experience with TB. For TB patients, this means when they were diagnosed, how they were diagnosed, how they access services. For others, this means how they’ve engaged with it through work.

*For TB patients:*

- When were you diagnosed with TB?
  - Probes: What test was used? Which sector did you access TB services?
- How long were you sick before you received TB care?
  - Probes: When did you start experience symptoms for TB? When did you seek care? When did you receive care?
- What barriers did you face when accessing TB care and services?
  - Probes: Can you easily get tested? Do you have funds to access services?
- What has helped you gain access to TB services (i.e. facilitators)?
  - Probes: Do you have transportation to a health center?
- Have you had any negative experiences from your diagnosis with TB?
  - Probes: Have you experienced stigma as a result of your diagnosis with TB? Have you lost your job from your diagnosis with TB?
- Have other people you are close with or live with had TB? How has that affected you?

*For public health officers and non-governmental staff:*

- How long have you worked in the TB field?
  - Probes: What do you do? What have you done?
- What do you value about working in the TB field?
  - Probes: How fulfilled are you in your work? How do you feel fulfilled in your work?
  - Note: asks about personal fulfillment working with Proper care

**Experience with Proper care (10 minutes)**

Use this section to explore their engagement with Proper care specifically.

*For TB patients:*

- When was your first contact with Proper care?
  - Probes: Did a CHW come to you? Tell me more about the services you accessed through Proper care.
- What other services do you access through Proper care?
  - Examples: social/financial support
- How long have you engaged with Proper care?
- How often do you engage with Proper care? (i.e. frequency)

*For public health officers and non-governmental staff:*

- What is your perception of Proper care?
  - Probes: in your own words, what is the purpose of Proper care?
- What is your role and engagement with Proper care?
  - Probes: Do you talk to TB patients? Do you do programmatic activities?
- Do you feel that Proper care is making an impact in society or in the lives of the TB patients? How?
  - Probes: what impact does Proper care have for TB patients? For you specifically? For your organization?

**Benefits and costs from Proper care (60 minutes)**

Use this section to explore their perceived benefits and costs from engagement with Proper care. This section will be divided into two parts: first is an open-ended discussion on perceived benefits from Proper care, then a concluding discussion on benefits. Second part is an open-ended discussion on perceived costs (i.e. obstacles and challenges) from Proper care, then a concluding discussion on these costs.

*Benefits (30 minutes):*

- **Open-ended discussion**: How do you think you have benefitted from engagement with Proper care? How have TB patients benefitted? How do you think others have benefitted from engagement with Proper care? How has society benefitted from Proper care?
  - Note: allow for open discussion and take note of benefits mentioned. Ask probing questions about each benefit mentioned (How does it benefit you? How does it benefit others? Tell me more about this benefit.)
- **Closed-ended discussion**: You’ve all mentioned ____, _____, and ______ (etc.) as benefits from engagement with Proper care. Let’s discuss each one more in-depth.
  - Ask participants to rank the benefits as a group, from most significant benefit to least significant benefit.

*Costs (30 minutes):*

- **Open-ended discussion**: What obstacles or challenges do you experience from engagement with Proper care? What obstacles or challenges do TB patients face from engagement with Proper care? What obstacles or challenges do others face from Proper care? What obstacles or challenges does society face from Proper care?
  - Notes: allow for open discussion and take note of the obstacles and challenges mentioned. Ask probing questions about each obstacle mentioned (How does it pose a challenge? Tell me more about this obstacle.)
- **Closed-ended discussion**: You’ve all mentioned ____, _____, and ______ (etc.) as obstacles or challenges from engagement with Proper care. Let’s discuss each one more in-depth.
  - Ask participants to rank the obstacles and challenges as a group, from most significant obstacle/challenge to least significant obstacle/challenge.

**Closure**

*We are coming to the end of our focus group now. Though there were many different opinions about _______, it appears unanimous that _______. Does anyone see it differently? It seems most of you agree ______, but some think that _____. Does anyone want to add or clarify an opinion on this?*

*Is there any other information regarding your experience with Proper care or TB that you think would be useful for me to know? Does anyone have any other questions?*

*Thank you very much for coming this afternoon. Your time is very much appreciated, and your comments have been very helpful. Your thoughts today will be kept confidential and anonymous in our analysis. You will be compensated for your time today (200,000 VND per person). We’ll turn off the tape recorder now.*

In-depth interview

**Participants**

- Notified TB patients detected through active case finding activities of the project
- Notified TB patients detected through passive case finding at the District TB Units
- Project support staff
- Site Coordinators (SCs)
- TB Counselors
- Community Health Works (CHWs)
- District TB Unit (DTU) and commune-level TB officers
- National TB Program (NTP) Representatives
- Province/District
- Donors/Sponsors

**Objectives**

- To explore the social and non-monetary benefits of Proper care
- To gather poignant quotes from TB patients, public health officers, and non-governmental staff on Proper care
- To expand on the focus group discussions (FGD) and delve deeper into the benefits and costs of Proper care
- To inform the SROI

**Introduction**

- Greet participant when they enter the door. Introduce FIT and study; confidentiality; timing

*Good afternoon. My name is ____ and this is my colleague ___. We are from FIT. Thank you for coming. An interview is a relaxed one-on-one conversation.*

*We are here today to talk about your experiences with the Proper care program. The purpose is to get your perceptions on how the program has benefitted you and society, especially in non-monetary ways.*

*There are no right or wrong answers. You can disagree and you can change your mind. I would like you to feel comfortable saying what you think and how you really feel.*

**Procedure:**

*_____ will be taking notes and tape recording the conversation so that I do not miss anything you have to say. I explained these procedures to you when we set up this meeting. As you know everything is confidential. You can decline to answer any question you do not feel comfortable answering and there will be no consequence for you. Any personal identification of interviewees will be anonymised. Feel free to stop me at any point and you do not have to answer anything you don’t want to. This conversation will last approximately one hour to 90 minutes. Would you like to proceed?*

**Participant Introduction (5 minutes)**

*Let’s start by sharing what you do, how long you’ve been diagnosed with TB (if in interview with TB patients), and how long you’ve been in/worked for the Proper care program.*

**Life/Work Background (5 minutes)**

Use this section to get a sense of the work that the staff does. For TB patients, use this section to explore more detailed circumstances of the TB patients and get a sense of their life and their social network.

*For TB patients*:

- Family background
  - Probes: Where were you born? Who is in your family? Tell me more about your family circumstances and household.
- School/education
  - Probes: What is your highest education level? Where did you go to school?
- Work history
  - Probes: What is your current work? How long have you been working? What other types of jobs have you had?
- Family relationships
  - Probes: How often do you interact with family?
- Community relationships
  - Probes: What social networks do you have outside of your family?

*For public health officers and non-governmental staff*:

- School/education
  - Probes: What is your highest education level? Where did you go to school?
- Work history
  - Probes: How long have you been working? What is your current work? What position do you hold? How long have you held this position? What are your main responsibilities? Tell me more about other positions/programs you worked with prior to this position.

**Experience with TB (5 minutes)**

Use this section to explore their experience with TB. For TB patients, this means when they were diagnosed, how they were diagnosed, how they access services. For others, this means how they’ve engaged with it through work.

*For TB patients*:

- When were you diagnosed with TB?
  - Probes: What test was used? Which sector did you access TB services?
- How long were you sick before you received TB care?
  - Probes: When did you start experience symptoms for TB? When did you seek care? When did you receive care?
- What barriers did you face when accessing TB care and services?
  - Probes: Can you easily get tested? Do you have funds to access services?
- What has helped you gain access to TB services (i.e. facilitators)?
  - Probes: Do you have transportation to a health center?
- Have you had any negative experiences from your diagnosis with TB?
  - Probes: Have you experienced stigma as a result of your diagnosis with TB? Have you lost your job from your diagnosis with TB?
- Have other people you are close with or live with had TB? How has that affected you?

*For public health officers and non-governmental staff:*

- How long have you worked in the TB field?
  - Probes: What do you do? What have you done?
- What do you value about working in the TB field?
  - Probes: How fulfilled are you in your work? How do you feel fulfilled in your work?
  - Note: asks about personal fulfillment working with Proper care

**Experience with Proper care (10 minutes)**

Use this section to explore their engagement with Proper care specifically.

*For TB patients:*

- When was your first contact with Proper care?
  - Probes: Did a CHW come to you? Tell me more about the services you accessed through Proper care.
- What other services do you access through Proper care?
  - Examples: social/financial support
- How long have you engaged with Proper care?
- How often do you engage with Proper care? (i.e. frequency)

*For public health officers and non-governmental staff:*

- What is your perception of Proper care?
  - Probes: in your own words, what is the purpose of Proper care?
- What is your role and engagement with Proper care?
  - Probes: Do you talk to TB patients? Do you do programmatic activities?
- Do you feel that Proper care is making an impact in society or in the lives of the TB patients? How?
  - Probes: what impact does Proper care have for TB patients? For you specifically? For your organization?

**Benefits and costs from Proper care (30-40 minutes)**

Use this section to explore their perceived benefits and costs from engagement with Proper care. Note to interviewer to probe more into answers – asking why? And how? And what happened next? Etc. Ask questions that participants are more comfortable answering in interviews compared to a group setting. This section builds on answers and questions from the FGD.

*For TB patients:*

- How do you think you have benefitted from engagement with Proper care?
  - Probes: Tell me more about these benefits.
  - Examples: increased knowledge, reduction in out-of-pocket expenditures, increased adherence, increased sense of self-efficacy, earlier detection
- Do you think Proper care has helped you save money on TB treatment? How so?
  - Probe: Do you borrow money to access TB treatment?
  - Note: asks about reduction in out-of-pocket expenditures
- Do you believe that you have a better knowledge on how to access TB treatment? How so? Do you have more confidence in knowing where to seek TB care?
  - Notes: asks about increased knowledge of TB and increased sense of self-efficacy
- Do you have increased knowledge of TB from Proper care? How so?
  - Notes: asks about increased knowledge of TB
- Do you have better adherence to TB prophylaxis from engagement with Proper care? How so?
  - Notes: asks about increased adherence from engagement with Proper care
- How do you think people feel about others with TB?
  - Probe: Would people eat or drink with friends who have TB? Would people talk with others who have TB? Do those with TB have difficulty finding jobs? Are people with TB afraid to tell their family they have TB? Do you or others with TB feel alone? Do you or others lose friends because of TB diagnosis? Are you or others with TB afraid of going to TB clinics because other people may see?
  - Note: asks about community and personal perception of TB stigma
- Have you felt healthier since engaging with Proper care?
  - Probe: Have you experienced less stigma and isolation since engaging with Proper care? Do you take your medication for TB as instructed since engaging with Proper care?
  - Examples: less stigma/isolation, increased treatment adherence
- What obstacles or challenges do you experience from engagement with Proper care?
  - Probes: Tell me more about these obstacles (i.e. taking time to get tested, diagnosed). Has your work been affected from going to appointments for TB or engaging with Proper care?
  - Examples: job loss, isolation and stigma

*For public health officers and non-governmental staff:*

- How do you think you have benefitted from engagement with Proper care?
  - Probes: Tell me more about these benefits. What skills have you gained from working with Proper care? How have you learned more about TB from working with Proper care?
  - Examples: employment, incentives, training/skill development, compensation, increased knowledge
- What challenges do you experience from engagement with Proper care?
  - Probes: Tell me more about these challenges. Have you experienced more pressure at work? Tell me more about this experience.
  - Examples: meeting targets, incentivization for targets, pressure from different organizations, increased reporting
- How does Proper care benefit society and TB patients? How does it benefit you specifically? How does it benefit your organization?
  - Probes: How does Proper care help bring more funding to your organization? How does it help your organization scale up TB systems? How does it decrease TB transmission?
  - Examples: macroeconomic productivity loss reduction, cost avoidance for treating TB, poverty reduction, increase TB funding, improve organization reputation, scale up systems, generate more scientific evidence, greater overall TB control, decrease TB transmission
- How does Proper care negatively affect society? How does it negatively affect you? How does it negatively affect your organization?
  - Probes: How does Proper care affect bureaucracy among TB organizations? How does Proper care exert more pressure on your work?
  - Examples: pressure, bureaucracy, compliance, not performing well because no communication

**Closure**

*We are at the end of our interview now.* *Is there any other information regarding your experience with Proper care or TB that you think would be useful for me to know? Do you have any other questions?*

*Thank you very much for coming this afternoon. Your time is very much appreciated, and your comments have been very helpful. Your thoughts today will be kept confidential and anonymous in our analysis. You will be compensated for your time today (200,000 VND per person). We’ll turn off the tape recorder now.*
