# Supplementary material for: Economic evaluation of a community health worker model for tuberculosis care in Ho Chi Minh City, Viet Nam: a mixed-methods Social Return on Investment Analysis
Source: BMC Public Health. 2023 May 25;23:945. doi: 10.1186/s12889-023-15841-2 (PMC10210363; doi:10.1186/s12889-023-15841-2)
Supplement: Supplementary file 3 — Supplementary Material 3 [file 12889_2023_15841_MOESM3_ESM.docx]

**Supplementary Information**

*Supplemental methods*

Rapid literature Review

We conducted a rapid literature review focusing on PubMed for evaluations of TB interventions and return on investment (ROI) analyses. Our search strategy was as follows:

| **Domain** | **Search terms** |
| --- | --- |
| SROI | Social return on investment OR SROI OR return on investment OR ROI |
| Cost-benefit analysis | Cost-benefit OR cost-benefit analysis OR cost-utility OR cost-utility analysis |
| Tuberculosis | Tuberculosis OR TB |
| Active case finding | Active case finding OR case finding OR ACF OR mobile case finding |
| Community health worker | Community health worker OR CHW OR community-based care |

Inclusion criteria were studies in any low- or middle-income country (LMIC) and anything published after 2000. Exclusion criteria were any studies in only abstract form and any studies with only qualitative results. Sample search terms included:

- ("return on investment"[MeSH Terms] OR ("return"[All Fields] AND "investment"[All Fields]) OR "ROI"[All Fields]) AND ("tuberculosis"[MeSH Terms] OR "tuberculosis"[All Fields])
- “tuberculosis”[MeSH Terms] OR “tuberculosis”[All Fields]) AND (“value driver”[MeSH Terms] OR (“value”[All Fields] AND “driver”[All Fields]) OR “value driver”[All Fields]) AND (value driver”[MeSH Terms] OR (“value”[All Fields] AND “driver”[All Fields]) OR “value driver”[All Fields])

From our initial search, we obtained 65 results and excluded 12 that were before the year 2000. We reviewed the abstracts of the remaining 53 and selected 12 for full review.

Additionally, we scanned grey literature through organizations such as the World Health Organization (WHO) and included two other papers in our review.
